# COVID-19 and Cardiovascular Diseases: A Literature Review From Pathogenesis to Diagnosis

**DOI:** 10.7759/cureus.35658

**Published:** 2023-03-01

**Authors:** Aroma Naeem, Shehroze Tabassum, Saima Gill, Maleeka Z Khan, Nimra Mumtaz, Qamoos Qaiser, Mubashar Karamat, Mashhood Arif, Farhan Naeem, Ahmed Afifi, Jawad Basit, Abdulqadir J Nashwan

**Affiliations:** 1 Internal Medicine, Mayo Hospital, Lahore, Lahore, PAK; 2 Medicine and Surgery, Lahore General Hospital, Lahore, PAK; 3 Internal Medicine, Aziz Fatimah Medical and Dental College, Faisalabad, PAK; 4 Medicine, Benha University, Benha, EGY; 5 Medicine, Holy Family Hospital, Rawalpindi, PAK; 6 Cardiology, Rawalpindi Medical University, Rawalpindi, PAK; 7 Nursing, Hamad Medical Corporation, Doha, QAT

**Keywords:** troponin, echocardiography, dysrhythmia, myocarditis, acute myocardial infarction, sars-cov-2, covid-19, cardiovascular insult, myocardial injuries

## Abstract

The coronavirus disease 2019 (COVID-19) took the world by storm after the first case of COVID-19 emerged in China on December 8, 2019. The disease is generally considered as an infection of the respiratory system, but serious life-threatening myocardial injuries have been reported with this infection. Coronavirus can damage cardiac myocytes by entering the cell through angiotensin-converting enzyme 2 (ACE-2) receptor binding. Myocardial infarction, myocarditis, heart failure, cardiac arrhythmias, and Takotsubo cardiomyopathy are cardiac clinical manifestations commonly seen among patients affected by COVID-19. These cardiac pathologies are seen both during ongoing infection and post-infection. Elevated levels of myoglobin, troponin, creatine kinase-MB, plasma interleukin-6, lactate dehydrogenase (LDH), and N-terminal pro-b-type natriuretic peptide (NT-proBNP) have been found in COVID-19-associated myocardial injuries. The diagnostic modalities used in myocardial injuries due to COVID-19 include electrocardiography (ECG), cardiac magnetic resonance imaging (CMR), endomyocardial biopsy, echocardiography (Echo), and computerized tomography (CT-Scan). This literature review will discuss, in detail, the pathogenesis, clinical manifestations, and diagnosis of myocardial injuries due to COVID-19.

## Introduction and background

The emergence of coronavirus disease 2019 (COVID-19), caused by Severe Acute Respiratory Distress Syndrome-Coronavirus-2 (SARS-CoV-2), has posed one of the greatest challenges to public health in the past few years. The contagious nature of this virion and high transmissibility, even during the asymptomatic phase, has contributed to making this disease a global pandemic. A province in China called Hubei reported the first victim of COVID-19 on December 8, 2019 [1]. Within a short span of three months, this infection managed to spread across 177 countries/territories and so forth became a global pandemic [1]. So far, millions of people have been afflicted by COVID-19, and the spread has not stopped till now. As of January 8th, 2023, the present number of confirmed cases of COVID-19 outbreak equates to 668,580,044, with 6,713,479 deaths worldwide [2]. In particular, the transmission mode via respiratory droplets has played a vital role in establishing it as a global pandemic. However, the primary system targeted by COVID-19 is the respiratory system. This disease's cardiovascular complications present alarming symptoms and can be life-threatening, especially among populations with co-existent cardiovascular diseases. According to recent data, mortality was higher among populations that developed COVID-19 along with acute myocardial injury as their underlying condition compared to those without any cardiac injury, corresponding to elevation in cardiac troponin (cTn) levels [3-4].

Important cardiovascular manifestations of COVID-19 include myocarditis, ischemic heart disease, and arrhythmias. Patients can present with non-specific symptoms of chest pain and palpitations [5]. Cardiac complications are common not only with COVID-19 infection, but recent studies have shown that these are reported with some mRNA COVID-19 vaccines as well, with effects ranging from cardiac inflammation to life-threatening thrombosis and myocardial ischemia [6]. Treatment options depend upon the extent of cardiac insult, ranging from mild therapy to ICU admissions. A better understanding of COVID-19 pathogenesis in causing cardiac clinical manifestations can be helpful in timely diagnosis and prompt therapy to lessen the potential harm, which can reduce cardiovascular mortality rates among individuals affected by COVID-19. Our article aims to provide a comprehensive review regarding pathogenesis, clinical manifestations, and diagnosis of myocardial injuries due to COVID-19.

## Review

Pathogenesis of myocardial injuries among COVID-19 patients

Role of Angiotensin-Converting Enzyme 2 (ACE-2)

Expression of ACE-2 is typically seen in cardiac myocytes and the cells of the respiratory system and other organ systems [7]. Viral glycoprotein spike 1 of SARS-CoV-2 binds ACE2 in the host cells [8]. It causes myocardial damage and, ultimately, downregulation of ACE2 and starts an inflammatory cascade, myocardial interstitial fibrosis, and coronary plaque destabilization [9-10]. ACE2 is involved in converting angiotensin II to vasodilator compound angiotensin 1. Blocking of the ACE2 enzyme results in the elevation of angiotensin II, which is a pro-inflammatory compound found to be associated with raised inflammatory cytokines in systemic circulation and considered to be associated with extrapulmonary manifestations of coronavirus infection such as cardiovascular disease [8].

Role of Cytokine Storm

Multiple studies have documented high levels of inflammatory markers and cytokines, including Interferon-gamma, Interleukin-1 beta (IL-1β), monocyte chemoattractant protein (MCP-1), IP-10, and TNF alpha, in patients of COVID-19 [11]. The potential contribution of cytokine storm in causing myocardial insult in COVID-19 patients can be divided into direct and indirect effects:

Direct effect of cytokine storm: A significant rise in MCP-1 is seen after the COVID-19 outset. It is important in the recruitment of monocytes/macrophages [12]. Recruitment of macrophages around the viral inclusions poses a major threat to the mechanical functioning of the heart. Histopathology showed abundant lymphocytes and macrophages in COVID-myocarditis patients [13]. IL-1β is another important cytokine in COVID-19 patients. It stimulates the release of other cytokines, including IL-21, IL-17, and IL-22, and thickens the heart layers, ultimately leading to cardiomyopathy [13-14].

Indirect effect of cytokine storm: Cytokine storm indirectly causes damage to the heart by its effect on the lungs leading to hypoxemia and decreased blood supply to cardiac vasculature [15].

Role of Coagulopathy

There is a great risk of thromboembolism among COVID-19 patients, which is depicted by higher levels of fibrin degradation products (FDPs) and D-dimers, particularly in seriously ill patients [16-17]. ACE2 expression on endothelial cells is a site of attack for the virus, leading to systemic endothelial damage. This systemic endothelium can lead to the activation of complement and thrombin systems and aggregation of white blood cells and platelets, collectively leading to coagulopathy [18]. Moreover, immobilization in seriously ill patients is another independent risk factor contributing to coagulopathy. The prothrombotic state can be followed by a blockage of the coronary arteries [19].

**Figure 1:**
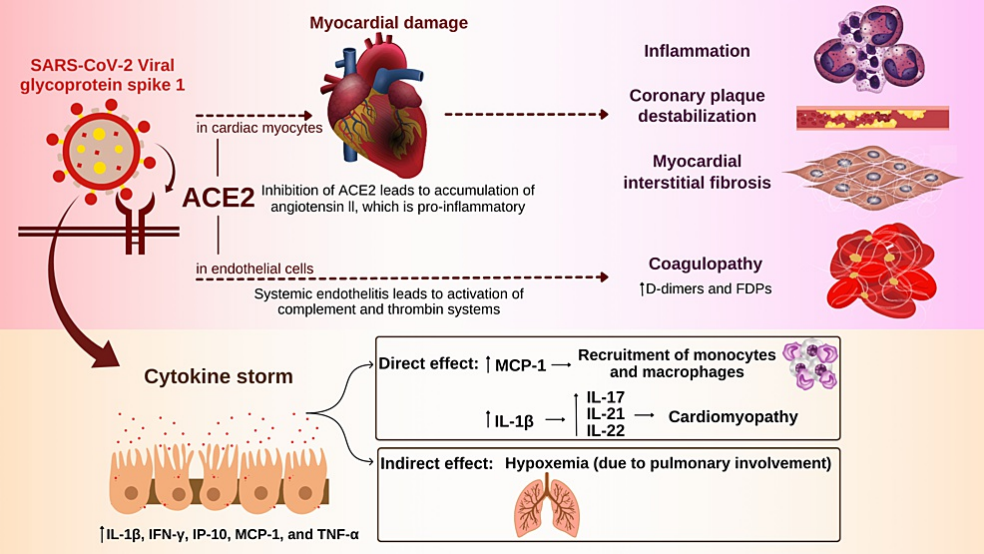
Illustrating pathogenesis involved in myocardial insult by COVID-19. Authors’ original work ACE-2: Angiotensin-converting enzyme 2; FDPs: Fibrin degradation products.

Libby and Lüscher [20] described COVID-19 as an endothelial illness, especially in its more advanced complex stages. It explains the pivotal role of the endothelium in thrombosis and fibrinolysis. The endothelium has vasodilator/vasoconstrictor balance, endothelial inflammatory balance, and antioxidant/pro-oxidant balance, along with the barrier function. Disturbance of these mechanisms in disease, along with the cytokine storm, has been hypothesized to be the main pathophysiological changes leading to endothelial damage. The pathogenesis of myocardial injuries in patients by COVID-19 is summarized in Figure 1.

Cardiac clinical manifestations of COVID-19

Myocardial Infarction

Myocardial infarction (MI) in COVID-19 is attributed to various pathological mechanisms. It is suggested that glycoproteins in the viral envelope can bind to both porphyrin and the β-chain of hemoglobin, leading to hypoxia which ultimately causes type 2 acute myocardial infarction (AMI), characterized by an imbalance in the myocardial oxygen demand due to infection and myocardial oxygen supply [9,20-21]. The pro-thrombotic state caused by the pro-inflammatory state can also aggravate Type 1 AMI [19,22].

Arrhythmias

Arrhythmias are another life-threatening complication identified in COVID-19 patients, which can result in both bradyarrhythmia and tachyarrhythmia [23]. The possible mechanism on top of direct cardiac damage by SARS-CoV-2 is electrolyte imbalances. For instance, hypokalemia due to renin-angiotensin-aldosterone system (RAAS) disturbance increases the risk of tachyarrhythmia [15,24]. Studies exploring the etiology of arrhythmias in COVID-19 patients with myocarditis have stated several causes, including direct cell injury, cell membrane rupture, ischemia, inflammatory cytokines, scarring, and pericarditis. Inflammatory cytokines result in dislodging of a desmosomal protein called plakoglobin and is the potent cause of arrhythmogenic cardiomyopathies [25].

Heart Failure

Heart failure is another clinical manifestation reported in COVID-19 patients [19]. Direct cardiac damage and a hyper-inflammatory state lead to necrosis in the myocardium. Moreover, endothelial insult and micro-thrombosis play a role in damaging the endocardium. These can eventually lead to the failure of the systolic and diastolic functioning of the heart, causing cardiogenic shock [13]. The compromised pulmonary vascular bed can lead to pulmonary hypertension and right heart failure [26].

Myocarditis

The direct cardiac damage and hyperinflammation caused by cytokine storms can lead to myocarditis. Viral inclusions lead to the recruitment of inflammatory cells, including monocytes, macrophages, neutrophils, and lymphocytes, which are associated with edema. Myocardial edema and necrosis of myocardial cells and connective tissue interstitium represent this viral-induced myocarditis. The cell-mediated auto-immune response caused by the virus can also lead to myocarditis [27]. Epicardial fat is linked with myocarditis, by acting as a reservoir for the COVID-19 virus and by the release of adipokines. The thickness of epicardial fat is directly associated with the extent of myocardial inflammation in COVID-19 patients [28].

Takotsubo Cardiomyopathy

Transient systolic and diastolic left ventricular dysfunction is a characteristic feature of this disease [29-30]. The disease is known to be preceded by an emotional and psychological trigger [31]. The role of catecholamines in its pathogenesis is widely studied and debated [32]. Studies have reported an increased incidence of this stress cardiomyopathy in COVID-19 patients [33]. The continuous stress, anxiety, fear, and panic attacks, in addition to the cytokine storm in COVID-19, can give rise to excessive catecholamine release that can trigger Takotsubo cardiomyopathy.

Myocardial Effects due to Post-COVID-19 Syndrome (PCS)

Pathogenesis in post-COVID-19 syndrome (PCS) includes autonomic nervous system (ANS) dysfunction. In the cardiovascular system, it manifests as chest pain, palpitations, orthostatic disorders, blood pressure variations, and rhythm disorders, including inappropriate sinus tachycardia (IST). IST is a prevailing condition among PCS patients and plays a vital role in cardiac symptoms seen in PCS, like palpitations, impaired exercise capacity, and fatigue [34].

Psychological Effects of COVID-19 on Heart

**Figure 2:**
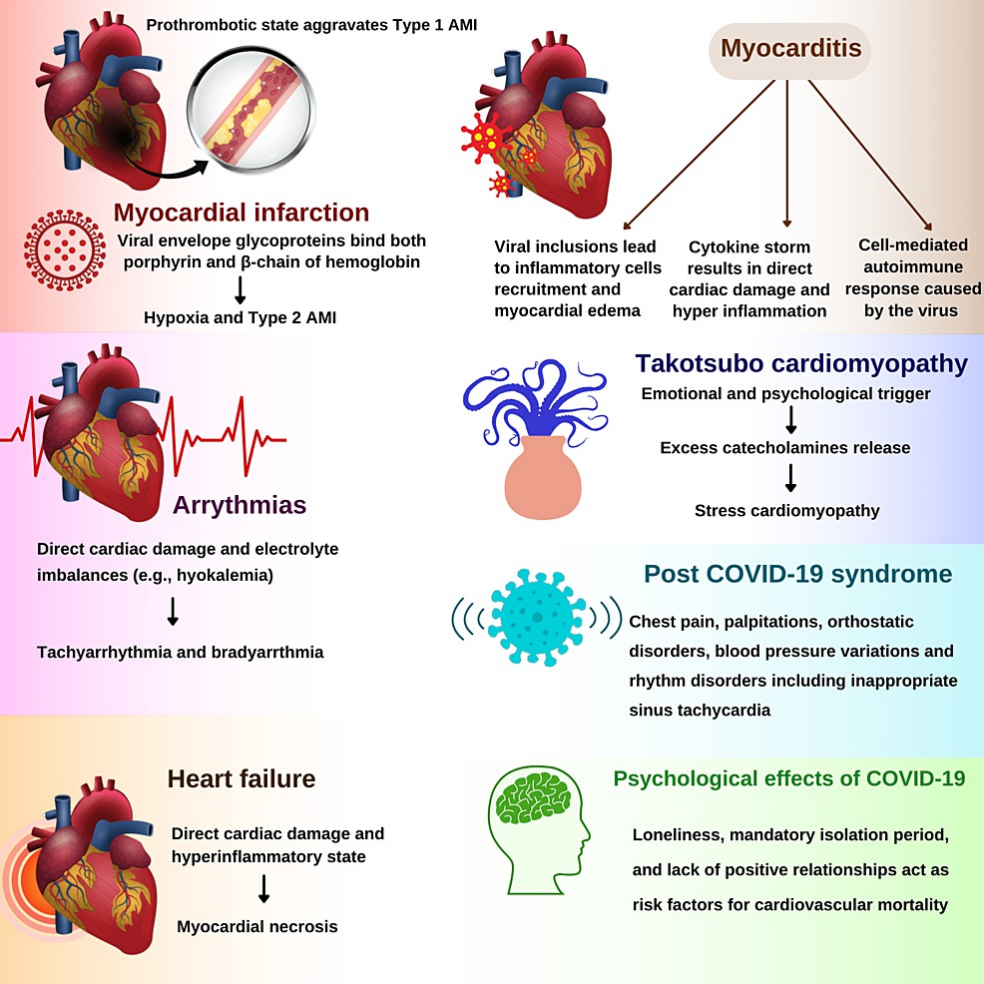
Illustrating myocardial clinical manifestations by COVID-19. Authors’ original work AMI: Acute myocardial infarction; COVID-19: Coronavirus disease of 2019.

Both clinical and experimental research has reported the cardiovascular consequences of social distancing. The impact of loneliness and mandatory isolation period on heart diseases are profound. The lack of positive relationships is recognized as an important risk factor for cardiovascular mortality [35-36]. Takotsubo cardiomyopathy due to psychological stress has already been discussed above. The emotional stressors that trigger Takotsubo cardiomyopathy are further increased by the isolation period, which needs to be followed by the patients of COVID-19 [33]. Cardiac clinical manifestations of COVID-19 are summarized in Figure 2.

Diagnosing cardiac injury among COVID-19 patients

In addition to routine laboratory testing, the role of various biomarkers in diagnosing COVID-19 is being investigated to diagnose COVID-19 with a low probability of misdiagnosis reliably. Biomarkers can assist clinicians in initiating treatment and monitoring COVID-19 patients. Elevated levels of different cardiac biomarkers have been found among COVID-19 patients [37]. Many reports have shown increased serum lactate dehydrogenase levels (LDH) in most COVID-19 patients. Elevated LDH levels have been linked with increased mortality in COVID-19 patients [38].

Levels of CK-MB are raised in COVID-19 patients with acute myocardial injury. Especially patients who did not survive the COVID-19 infection were found to have raised CK-MB levels. However, raised myoglobin levels are more specific for cardiac insult in COVID-19 patients [39].

Plasma concentrations of interleukin-6 are elevated in COVID-19 patients with cardiac insult [5]. According to one meta-analysis, patients with complicated diseases had 2.9 times higher mean interleukin-6 levels than those with uncomplicated diseases [40].

Prothrombotic endothelial injury due to COVID-19 increases the likelihood of venous thromboembolism, acute myocardial infarction, and pulmonary embolism. In COVID-19 patients, raised D-dimers and fibrin degradation products indicate the development of disseminated intravascular coagulation and pulmonary embolism, which impact hemodynamic stability. Elevated D-dimer levels in COVID-19 patients have also been associated with higher mortality rates than those without a rise in D-dimers [38].

Troponin levels are elevated due to myocardial injury and myocarditis, which may be due to an increased workload on the heart, poor oxygen supply, or direct tissue injury. The raised troponin level in COVID-19 patients is a direct risk for severe outcomes such as death. In sepsis, troponin levels were also found to be of prognostic significance. However, a negative troponin profile does not exclude myocardial injury. High-sensitivity Troponin I (hs-TnI) is more reliable for diagnosing myocardial injury [38-39].

Elevated NT‐proBNP levels have been found in cardiac involvement in COVID-19 patients. NT-proBNP or BNP levels may also be elevated in patients who show no signs or symptoms of fluid overload, regardless of myocardial injury [41].

Other biomarkers of systemic inflammation, such as erythrocyte sedimentation rate (ESR), C-reactive protein (CRP), glucose, neutrophils, and neutrophil/lymphocyte ratio, are found to be increased in COVID-19 patients with myocardial injury. However, these biomarkers may be non-specific and less sensitive [28].

Electrocardiography

In COVID-19 patients with myocarditis, electrocardiography (ECG) may show ST elevation and PR depression, indicating pericardial injury. However, QT interval prolongation, arrhythmia, bundle branch block, and premature ventricular complexes may also be depicted on ECG [26]. Cardiac complications from COVID-19 recorded on ECG may also reveal T wave inversion and non-specific ST segment disruption [42]. ECG may reveal some patients' de-novo development of fragmented QRS complexes [43]. ECG can be considered as the screening test for myocardial complications caused by COVID-19.

Echocardiography and Cardiac Magnetic Resonance Imaging

Echocardiography (Echo) and cardiac magnetic resonance imaging (CMR) are recommended by American Heart Association (AHA) to confirm myocarditis in COVID-19 patients [44]. Although endomyocardial biopsy (EMB), as a diagnostic test, is considered a gold standard for confirmation of myocarditis. However, myocardial involvement in viral myocarditis is focal and patchy, making EMB less sensitive. This necessitates non-invasive imaging tests as an essential part of the workup. Hence, CMR is the most beneficial test for viral myocarditis [45]. In a study, CMR showed myocardial inflammation, edema, and scarring in individuals with COVID-19-related myocarditis. Although CMR is a better technique than ECHO, it is less recommended because of the need for decontamination after usage and slower output. Echocardiography is recommended as it can indicate myocardial injury due to acute coronary syndrome (ACS) as a wall defect [46]. The limitations of CMR include limited availability, high cost, extended exam time, and patient-specific issues like arrhythmia, difficulty in holding one's breath, claustrophobia, implanted metallic devices, and contrast hypersensitivity [47].

CMR is the key diagnostic test for the diagnosis of acute myocarditis in the context of stable patients. Echocardiography, as opposed to CMR, enables us to perform a bedside diagnostic and prognostic assessment, allowing us to circumvent challenges associated with transporting COVID-19 patients who are unstable [48].

Endomyocardial Biopsy (EMB)

The AHA and the European Society of Cardiology presented endomyocardial biopsy (EMB) as the quintessential diagnostic aid for myocarditis [49]. EMB is considered the gold standard investigation for confirmation of myocarditis [45]. In COVID-myocarditis patients, EMB revealed an abundance of lymphocytic infiltrate and macrophages [47]. EMB can also supply tissues to look for biomarkers for a more accurate diagnosis of COVID-related myocarditis. However, EMB has its limitations, including low sensitivity, required expertise, and risk of COVID-19 spread. Therefore, if invasive right heart catheterization is required, EMB should be undertaken concurrently to reduce the risk of contagious spread [25].

EMB is an invasive procedure that should only be used in life-threatening clinical scenarios where histological information might direct therapy decisions [48].

Computerized Tomography (CT-Scan)

In a study by Özer et al. [27], the computerized tomography (CT-Scan) technique was used to evaluate the thickness of epicardial fat. Epicardial fat acts as a reservoir of coronavirus and contributes to cytokine-mediated myocardial inflammation among patients with COVID-19.

**Figure 3:**
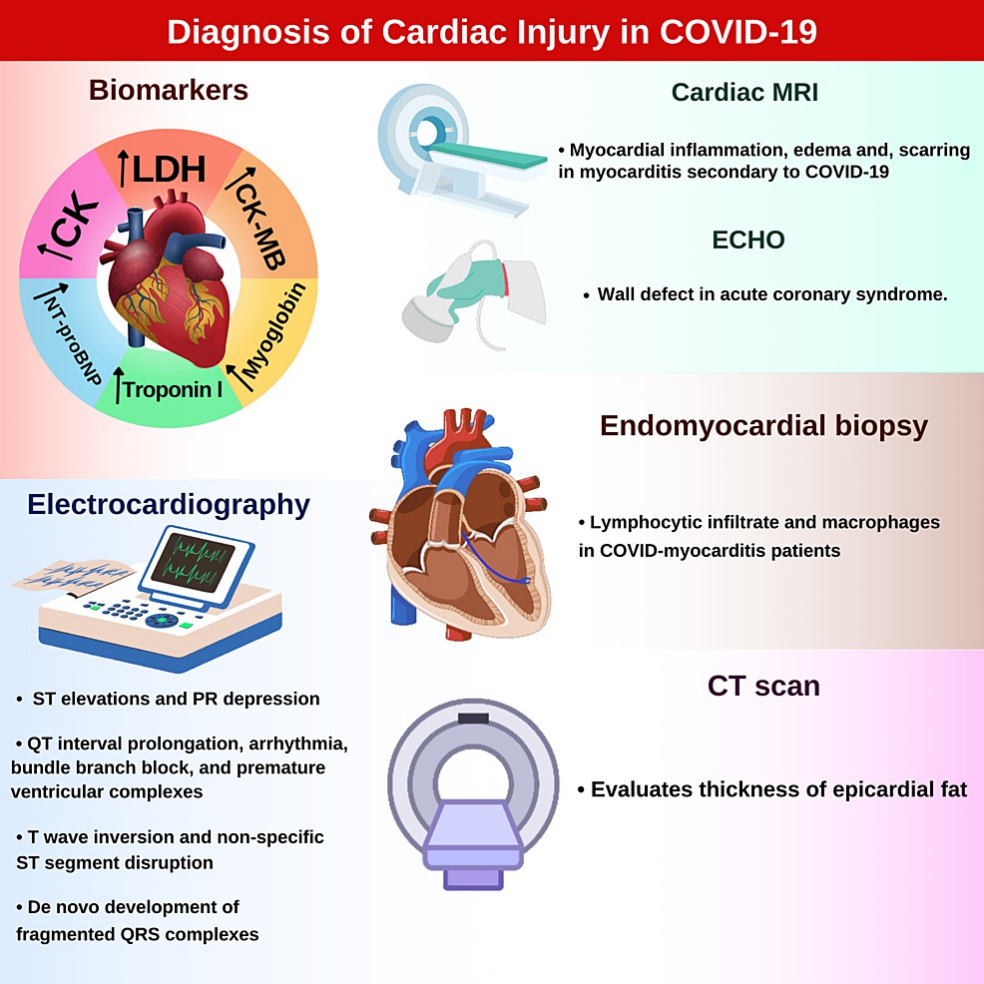
Illustrating modalities for diagnosing myocardial injuries due to COVID-19. Authors’ original work COVID-19: Coronavirus disease of 2019; LDH: Lactate dehydrogenase; CK-MB: Creatine kinase-myocardial band; NT-proBNP: N-terminal pro-b-type natriuretic peptide; CK: Creatine kinase; MRI: Magnetic resonance imaging; Echo: Echocardiogram; COVID: Coronavirus disease; CT: Computed tomography.

Bihan et al. found that the mean epicardial adipose tissue volume was linked to death or transfer from a non-ICU hospital medical department to an ICU in the selected cohort with a high proportion of obese individuals affected with COVID-19. To accurately predict the prognosis of COVID-19, measuring epicardial fat volume upon hospitalization may be helpful [50]. Diagnostic modalities used in myocardial injuries due to COVID-19 are summarized in Figure 3.

## Conclusions

COVID-19 and its associated injuries have been incriminated with many severe consequences, even death, which makes this concern a public health issue. Myocardial injury remains a significant sequela of COVID-19-induced insults. Scientific investigations are ongoing regarding the underlying pathophysiology and clinical manifestations to ultimately disentangle the complex mechanisms involved. The role of ACE2, cytokine storm, endothelial damage, and coagulopathy are some of the major factors leading to the cardiac manifestations of COVID-19. Several diagnostic modalities such as ECG, Echo, CMR, EMB, and CT scan are used for detecting the pathological changes caused by COVID-19. Several studies have been published on this topic since the pandemic's beginning, but further studies are required to strengthen the evidence.
